# DKK1 Promotes Cisplatin Resistance in Oral Squamous Cell Carcinoma via SLC3A2‐Dependent Ferroptosis Suppression

**DOI:** 10.1002/cnr2.70715

**Published:** 2026-10-08

**Authors:** Zhuoxuan Liu, Xiaolan Chen, Yucong Lin, Lijun Zhu

**Affiliations:** ^1^ Department of Oral and Maxillofacial Surgery, Guangdong Provincial People's Hospital (Guangdong Academy of Medical Sciences) Southern Medical University Guangzhou China; ^2^ Department of Stomatology The Second People's Hospital of Panyu Guangzhou Guangzhou China; ^3^ Orthopedic Center, The Third Affiliated Hospital of Guangzhou Medical University Guangzhou China

**Keywords:** cisplatin resistance, DKK1, ferroptosis, oral squamous cell carcinoma, SLC3A2

## Abstract

**Background:**

Oral squamous cell carcinoma (OSCC) is a prevalent malignancy associated with poor outcomes, largely attributable to cisplatin resistance—the standard chemotherapeutic agent.

**Aims:**

While ferroptosis has been proposed as a promising therapeutic target, how it is regulated in OSCC remains largely unclear.

**Methods and Results:**

To determine DKK1 expression levels and their prognostic implication, the TCGA‐HNSC cohort and OSCC patient samples were examined. The impact of DKK1 silencing and/or cisplatin treatment on cell proliferation and ferroptosis‐related markers, including reactive oxygen species (ROS), malondialdehyde (MDA), and glutathione (GSH) levels, was investigated in vitro. Additionally, the efficacy of DKK1 inhibition was evaluated in xenograft models. The findings revealed that DKK1 expression was markedly elevated in the TCGA‐HNSC cohort and was further validated in OSCC tissues. High DKK1 expression was associated with worse survival and contributed to cisplatin resistance, while DKK1 silencing sensitized cells to ferroptosis and enhanced cisplatin cytotoxicity. Mechanistically, our data support a regulatory model in which DKK1 maintains SLC3A2 expression through modulation of the β‐catenin/STAT3 axis, thereby contributing to ferroptosis suppression and cisplatin resistance. In vivo, DKK1 inhibition improved cisplatin efficacy, at least in part, by promoting SLC3A2‐associated ferroptosis.

**Conclusion:**

These findings support DKK1 as a potential regulator of ferroptosis and cisplatin resistance in OSCC. Targeting the DKK1/SLC3A2 axis to restore ferroptotic sensitivity may represent a promising strategy to overcome chemoresistance.

**Abbreviations**
CAFs: cancer‐associated fibroblasts
CR: cisplatin‐resistant
DDP: cisplatin
DKK1: Dickkopf‐1
GSH: glutathione
IHC: immunohistochemical staining
MDA: malondialdehyde
OS: overall survival
OSCC: oral squamous cell carcinoma
ROS: reactive oxygen species
RT‐qPCR: reverse transcription‐quantitative PCR
TCGA: the Cancer Genome Atlas
TME: tumor microenvironment

## 1 Introduction

As the leading type of oral cavity cancer, oral squamous cell carcinoma (OSCC) represents over 90% of all oral cancer cases and contributes significantly to the global disease burden [1, 2]. Based on the 2022 global epidemiological landscape, cancers of the lip and oral cavity constitute nearly 380 000 new diagnoses and 180 000 mortalities each year, making it the 18th most prevalent and lethal malignancy worldwide [3]. While surgical and medical interventions have progressed, patients confronting advanced OSCC still endure a grim prognosis, facing a 5‐year survival rate of less than half. This is largely due to late‐stage diagnosis, high recurrence rates, and treatment resistance [4]. Platinum‐based chemotherapy, especially cisplatin, remains the cornerstone of systemic treatment for locally advanced and recurrent/metastatic OSCC [5]. Cisplatin primarily exerts its antitumor effects by forming DNA adducts, leading to apoptosis. However, intrinsic or acquired resistance commonly compromises clinical effectiveness, lowering response to therapy and contributing to unfavorable patient survival [6]. The mechanisms underlying cisplatin resistance in OSCC are multifaceted, including enhanced DNA damage repair, altered drug uptake and efflux, evasion of apoptosis, and dysregulated cell death pathways [6]. Thus, identifying the molecular drivers of cisplatin resistance is crucial for developing new therapies to enhance OSCC outcomes.

Ferroptosis is an iron‐dependent form of regulated cell death driven by excessive lipid peroxidation and subsequent membrane damage [7, 8]. In OSCC, ferroptosis has received increasing attention because oral cancer cells are exposed to high oxidative stress and metabolic remodeling, making them potentially vulnerable to perturbations in redox homeostasis [9]. Current studies indicate that ferroptosis is controlled by interconnected regulatory networks, including iron metabolism, lipid peroxide generation, the system Xc−/GSH/GPX4 antioxidant axis, and alternative ferroptosis‐defense pathways such as FSP1‐related mechanisms [9, 10]. These pathways are also clinically relevant, as ferroptosis‐related genes have been explored as prognostic biomarkers and therapeutic targets in OSCC [9]. Importantly, accumulating evidence suggests that ferroptosis evasion contributes to cisplatin resistance in OSCC and head and neck cancer models. Inhibition of system Xc−/SLC7A11 or related antioxidant pathways can enhance cisplatin sensitivity, whereas activation of redox‐defense programs such as Nrf2/HO‐1/xCT signaling may protect resistant cells from ferroptotic death [10, 11, 12]. Recent studies have further linked AEBP1/MAPK signaling and circRNA‐miRNA‐SLC7A11 regulatory networks to ferroptosis‐mediated chemoresistance in oral cancer [13, 14]. However, most available studies have focused on intracellular ferroptosis executors or downstream antioxidant components, whereas how upstream tumor‐promoting factors regulate system Xc− associated ferroptosis resistance in cisplatin‐resistant OSCC remains incompletely understood. This knowledge gap limits the development of targeted strategies to restore ferroptotic vulnerability and overcome treatment resistance.

Dickkopf‐1 (DKK1), a secreted glycoprotein and canonical antagonist of the Wnt/β‐catenin signaling pathway, plays a critical role in embryonic development, bone homeostasis, and tumor biology [15]. Previous studies have linked abnormal DKK1 levels to a range of malignancies—hepatocellular carcinoma, breast cancer, multiple myeloma, and OSCC among them [16, 17, 18, 19]. Elevated DKK1 levels promote tumor progression by modulating cell proliferation, invasion, and immune evasion, often through Wnt‐dependent mechanisms, but also via Wnt‐independent pathways involving PI3K/AKT, NF‐κB, and STAT3 signaling [20, 21, 22, 23]. Emerging evidence suggests that DKK1 significantly contributes to resistance across multiple treatment paradigms: cytotoxic chemotherapy, targeted therapy, and immunotherapeutic approaches. Gastric cancer studies have shown that high DKK1 levels confer cisplatin resistance through PI3K/AKT pathway activation and EMT promotion [24]. In hepatocellular carcinoma, DKK1 enhances sorafenib resistance by inhibiting PI3K/AKT and Wnt/β‐catenin pathways [25]. Additionally, DKK1 facilitates paclitaxel resistance in breast cancer by suppressing CD8+ T cell infiltration and activity [26]. DKK1 secretion into the tumor microenvironment (TME) also suppresses antitumor immunity, contributing to resistance against immune checkpoint inhibitors [27]. Notably, targeting DKK1 with neutralizing antibodies or small molecules has been shown to restore drug sensitivity in preclinical models [28]. Despite progress, how DKK1 influences ferroptosis when cells become chemo‐resistant is still largely a mystery. Dissecting this regulatory mechanism holds promise for developing novel interventions against cisplatin resistance in OSCC.

This study investigated how DKK1 drives cisplatin‐induced ferroptosis resistance in OSCC. Analysis of the TCGA‐HNSC cohort and validation in OSCC patient samples indicated that DKK1 was upregulated in tumor tissues. Elevated DKK1 expression strongly correlated with poor overall survival (OS), highlighting its prognostic significance. Functional assays demonstrated that increased DKK1 expression confers cisplatin resistance, as evidenced by the generation of cisplatin‐resistant OSCC cell lines. Mechanistically, DKK1 was found to suppress ferroptosis by upregulating SLC3A2 expression through the Wnt/β‐catenin/STAT3 signaling axis. Furthermore, in vivo experiments showed that DKK1 inhibition potentiated cisplatin efficacy by promoting SLC3A2‐mediated ferroptosis. These observations indicate that DKK1 is central to determining therapeutic outcomes, which positions DKK1 as an attractive candidate for intervention to combat drug‐resistant OSCC.

## 2 Materials and Methods

### 2.1 Clinical Specimens

Twelve paired OSCC specimens and their corresponding non‐tumorous adjacent tissues were procured from patients treated at Guangdong Provincial People's Hospital (Guangdong Academy of Medical Sciences), Southern Medical University (Guangzhou, China). This research was conducted in strict compliance with the ethical guidelines of the Declaration of Helsinki. Institutional Ethics Committee clearance was granted (approval no. KY2025‐737‐02), and all enrolled subjects provided formal written informed consent prior to surgery. The clinical cohort comprised seven males and five females (median age: 65 years; range: 41–83 years). Based on the AJCC TNM classification, the tumors were equally distributed between early (stage I–II, *n* = 6) and advanced (stage III–IV, *n* = 6) stages. Detailed clinicopathological profiles are summarized in Table SI.

### 2.2 Bioinformatics Analyses

Transcriptomic profiles (FPKM format) and their corresponding clinical metadata were downloaded from the TCGA repository. Following log2 transformation of the RNA‐seq data, differential expression profiling was executed utilizing the R “limma” package (version 4.x), with *p*‐values subjected to Benjamini–Hochberg False Discovery Rate (FDR) correction. To evaluate gene co‐expression patterns, Spearman's rank correlation was applied, generating correlation coefficients (*ρ*) and adjusted statistical significance. All data preprocessing, normalization, and graphical output were carried out in R utilizing the limma, edgeR, ggplot2, and corrplot packages. A two‐sided *p*‐value of < 0.05 was considered statistically significant unless stated otherwise.

### 2.3 Immunohistochemical Staining (IHC)

IHC was performed following standard protocols. Following formalin fixation and paraffin embedding, tissue blocks were cut into 4‐μm sections. These sections were then probed with DKK1‐specific antibodies (Proteintech, Cat# 21112‐1‐AP; 1:200 dilution). Rabbit IgG was used as a negative control, and previously confirmed positive specimens were used as controls. Staining was evaluated based on both extent (0: none; 1: ≤ 10%; 2: 11%–50%; 3: 51%–75%; 4: > 75%) and intensity (1+: weak; 2+: moderate; 3+: strong). The immunoreactivity score (S) was calculated by combining the proportion of positive cells (*P*) and staining intensity (*I*) using the equation *S* = *P* × *I*, with localization assessed in the nucleus, cytoplasm, or membrane.

### 2.4 Reverse Transcription‐Quantitative PCR (RT‐qPCR)

Cellular and tissue‐derived RNA isolation was performed applying the TRIzol reagent protocol (Thermo Fisher Scientific Inc.). Subsequently, 2 μg aliquots of the purified RNA were converted into cDNA utilizing the High‐Capacity cDNA Reverse Transcription Kit (Applied Biosystems). SYBR Green PCR Master Mix (Thermo Fisher Scientific Inc.) and the Applied Biosystems 7500 Real‐Time PCR System were used to quantify gene expression. Table SII provides the primer sequences.

### 2.5 Western Blot Analysis

After extracting proteins using RIPA buffer containing a protease inhibitor mix, we quantified each sample via the BCA assay (Beyotime, cat. no. P0012). The concentrations were then normalized for subsequent loading. Equal amounts of protein were separated by SDS–PAGE and transferred to PVDF membranes (MilliporeSigma). Primary antibodies (Table SIII) were applied to the membranes overnight at 4°C, followed by HRP‐conjugated secondary antibodies for 1 h at room temperature. GAPDH served as a loading control. Immunoreactive bands were detected using an enhanced chemiluminescence system (ECL; Beyotime Institute of Biotechnology) and quantified by densitometry, with signals normalized to GAPDH.

### 2.6 Cell Culture and Reagents

The human OSCC cell lines CAL‐27 and SCC‐9 were obtained from Procell Life Science & Technology Co. Ltd. (Wuhan, China). Cell identity was confirmed by short tandem repeat (STR) profiling. All cell lines were routinely tested for mycoplasma contamination using a Mycoplasma Detection Kit, and only mycoplasma‐negative cells were used for experiments. Cells were maintained at 37°C in a humidified incubator with 5% CO_2_ and cultured in high‐glucose DMEM supplemented with 10% fetal bovine serum (FBS) and 1% penicillin–streptomycin (Solarbio Science & Technology Co. Ltd.). For STAT3 inhibition experiments, cells were treated with the STAT3 inhibitor **STAT3‐IN‐3** (1 μM; cat. no. HY‐128588; MCE) for 24 h.

### 2.7 Establishment of Cisplatin‐Resistant Cell Lines

Baseline cisplatin sensitivity was first established by calculating the half‐maximal inhibitory concentration (IC50) in wild‐type CAL‐27 and SCC‐9 cell lines via a 72‐h CCK‐8 viability assay across escalating drug doses. To establish chemoresistant models, the parental populations underwent continuous exposure to step‐wise increments of cisplatin (Selleck Chemicals) over a 3–6‐month period. The induction process initiated at 1 μM, with media replaced every 48 h. Once established, these resistant sublines (CAL‐27‐CR and SCC‐9‐CR) were continuously cultured in the presence of 10 μM cisplatin to guarantee the stability of their chemoresistant phenotype.

### 2.8 Establishment of DKK1 Stable Knockdown Cell Lines

To generate stable DKK1‐knockdown models, cisplatin‐resistant CAL‐27 and SCC‐9 cells were transduced with lentiviral particles encoding two validated shRNAs (shDKK1‐#1, 5′‐CGGTTCTCAATTCCAACGCTA‐3′; shDKK1‐#2, 5′‐CCTGTCCTGAAAGAAGGTCAA‐3′) or a non‐targeting control (shCtrl, 5′‐CCTAAGGTTAAGTCGCCCTCG‐3′) in the pLKO.1‐puro vector. To produce lentiviral particles, HEK293T cells were co‐transfected with shRNA plasmids, psPAX2, and pMD2.G via Lipofectamine 3000 (Thermo Fisher Scientific Inc.). At 48 and 72 h post‐transfection, virus‐containing supernatants were collected, filtered (0.45 μm), and added to CAL‐27‐CR and SCC‐9‐CR cells along with 8 μg/mL polybrene. Following transduction, we applied puromycin (3 μg/mL) for 7 days to enrich positive cells. Knockdown efficiency was validated by RT‐qPCR and western blot analysis.

### 2.9 Cell Viability Assay

To evaluate cell proliferation and survival, the Cell Counting Kit‐8 (CCK‐8; Beyotime, cat. no. C0037) was employed in strict accordance with the manufacturer's protocol. Briefly, 96‐well culture plates were seeded with cellular suspensions at a density of 3–5 × 10^3^ cells/well and left overnight to allow for firm adherence. Post‐treatment, a mixture containing 10 μL of CCK‐8 reagent and 100 μL of fresh culture medium was introduced into each well, followed by an additional incubation period of 1 to 4 h at 37°C. Absorbance at 450 nm was recorded on a BioTek microplate reader. All conditions were performed with at least six replicates. Viability was calculated using the formula: (OD_treatment − OD_blank)/(OD_control − OD_blank) × 100%.

### 2.10 Malondialdehyde (MDA) Assay

MDA levels were evaluated using the MDA Assay Kit (Beyotime, cat. no. S0131) with slight modifications. Cells (1 × 10^6^) seeded in 10‐cm dishes were treated for 24 h and harvested in 200 μL ice‐cold lysis buffer. Following centrifugation at 12 000*g* for 10 min at 4°C, supernatants were collected for both the MDA assay and protein quantification (BCA). Samples were mixed with thiobarbituric acid (TBA), incubated at 100°C for 15 min, chilled on ice, and centrifuged at 1000*g* for 10 min. Absorbance at 532 nm was recorded, and MDA content was expressed as nmol/mg protein after normalization.

### 2.11 Glutathione (GSH) Assay

Measurement of total GSH content was performed with the GSH Assay Kit (Beyotime, cat. no. S0052) following the supplier's recommended procedure. Specimens were homogenized in chilled assay buffer and centrifuged at 10 000*g* for 10 min at 4°C. The reaction was triggered by the addition of NADPH to a solution containing supernatant, DTNB, and glutathione reductase. Absorbance at 412 nm was measured with a microplate reader (Molecular Devices LLC), and GSH concentrations were derived from a standard curve, then normalized to protein content.

### 2.12 Measurement of ROS Levels

Intracellular ROS were measured using the CellROX Oxidative Stress Reagent (Thermo Fisher Scientific Inc.). Cells were rinsed with PBS and exposed to 10 μM CellROX in serum‐free medium for 30 min at 37°C in the dark. Fluorescence was recorded on a microplate reader or imaged via fluorescence microscopy (Leica DMi8). For quantitative analysis, fluorescence intensity was normalized to viable cell counts obtained from parallel CCK‐8 assays.

### 2.13 Flow Cytometry Analysis

Following a 72‐h transfection period, cells were pelleted, washed in PBS, and finally taken up in binding buffer. Staining was performed using the Annexin V‐FITC/PI Apoptosis Detection Kit (Invitrogen, Thermo Fisher Scientific, cat. no. V13242) under dark conditions for 30 min. For validation of cisplatin resistance, parental and CR cells were treated with 10 μM cisplatin before Annexin V/PI staining. Apoptosis rates were quantified using a flow cytometer and analyzed using FlowJo software (version 10.6; FlowJo LLC).

### 2.14 Animal Experiments

All in vivo experimental protocols received formal clearance from the Animal Ethics Committee of Guangdong Provincial People's Hospital (approval no. KY2025‐737‐02). To establish the murine xenograft models, 5‐week‐old BALB/c nude mice received subcutaneous inoculations into their right flanks. The injections consisted of 5 × 10^6^ CAL‐27‐CR cells (stably expressing shCtrl, shDKK1, or shDKK1 + OE‐SLC3A2) suspended in 100 μL of PBS. Tumor dimensions were recorded every 3 days, and the corresponding volumes were estimated utilizing the standard equation: Volume = (length × width^2^)/2. When tumors reached a volume of ~100 mm^3^, mice were randomly assigned to groups and treated with cisplatin (2.5 mg/kg, twice a week, intraperitoneally) or vehicle control. Cisplatin is a classical chemotherapeutic agent, and this dosage is widely established as the standard therapeutic window for OSCC xenograft models, providing effective tumor inhibition with manageable toxicity [29, 30]. Animals were weighed periodically throughout the treatment phase. At the study endpoint, mice were anesthetized via an intraperitoneal injection of sodium pentobarbital (50 mg/kg) to minimize suffering, and subsequently euthanized by cervical dislocation. Death was confirmed by the cessation of heartbeat and respiration. Following confirmation of death, tumors were excised and weighed. The maximum tumor diameter observed in the study was 14.1 mm, and the maximum tumor volume was 1020.9 mm^3^, which remained within the ethically approved limits for tumor burden.

### 2.15 4‐HNE Staining

For ferroptosis evaluation in xenograft tissues, IHC staining was performed using antibodies against 4‐HNE. Staining intensity and positive area were quantified using ImageJ, and IHC scores were calculated based on staining intensity and the percentage of positive tumor cells.

### 2.16 C11‐BODIPY 581/591 Staining

Lipid peroxidation was assessed using C11‐BODIPY 581/591 staining. Briefly, treated CAL‐27‐CR and SCC‐9‐CR cells were washed twice with PBS and incubated with C11‐BODIPY 581/591 probe at a final concentration of 5 μM in serum‐free medium for 30 min at 37°C in the dark. After staining, cells were washed with PBS to remove excess probe. Fluorescence images were captured using a fluorescence microscope. Lipid peroxidation was evaluated based on the increase in oxidized C11‐BODIPY fluorescence, and data were normalized to the corresponding control group.

### 2.17 FerroOrange Staining

Intracellular ferrous iron (Fe^2+^) levels were detected using FerroOrange staining. Briefly, treated CAL‐27‐CR and SCC‐9‐CR cells were washed twice with PBS and incubated with FerroOrange probe at a final concentration of 1 μM in serum‐free medium for 30 min at 37°C in the dark. After incubation, cells were washed with PBS and immediately observed under a fluorescence microscope. Fluorescence intensity was quantified using ImageJ software and normalized to the corresponding control group.

### 2.18 Chromatin Immunoprecipitation‐qPCR (ChIP‐qPCR)

ChIP‐qPCR was performed to evaluate STAT3 enrichment at the SLC3A2 promoter region in CAL‐27‐CR cells. Briefly, cells were cross‐linked with 1% formaldehyde for 10 min at room temperature and quenched with glycine. After cell lysis, chromatin was sheared into DNA fragments by sonication. The fragmented chromatin was immunoprecipitated overnight at 4°C using an anti‐STAT3 antibody or normal IgG as a negative control. Immune complexes were collected with protein A/G magnetic beads, followed by washing, elution, and reversal of cross‐linking. Purified DNA was analyzed by qPCR to quantify STAT3 enrichment at the SLC3A2 promoter region. Enrichment was calculated relative to input chromatin and normalized to the IgG control.

### 2.19 Dual‐Luciferase Reporter Assay

The SLC3A2 promoter region containing the predicted STAT3‐binding site was cloned into a firefly luciferase reporter vector. Stable shCtrl and shDKK1 CAL‐27‐CR cells were seeded in 24‐well plates and co‐transfected with the SLC3A2 promoter reporter plasmid and a Renilla luciferase plasmid as an internal control. Where indicated, cells were treated with the STAT3 inhibitor STAT3‐IN‐3 (1 μM) for 24 h before luciferase activity measurement. Firefly and Renilla luciferase activities were measured using a dual‐luciferase reporter assay system according to the manufacturer's instructions. Relative promoter activity was calculated by normalizing firefly luciferase activity to Renilla luciferase activity.

### 2.20 Statistical Analysis

Quantitative data are presented as the mean ± standard deviation (SD). Statistical analyses were performed using SPSS version 17.0 (SPSS Inc.) and GraphPad Prism software. Normality was assessed using the Shapiro–Wilk test, and homogeneity of variance was evaluated using Levene's test. For comparisons between two groups, normally distributed data with equal variance were analyzed using Student's *t*‐test, with paired tests applied where appropriate. For comparisons among three or more groups, one‐way analysis of variance (ANOVA) followed by Tukey's post hoc test was used when the assumptions of normality and equal variance were met. For data that did not meet these assumptions, non‐parametric tests, including the Mann–Whitney *U* test or Kruskal‐Wallis test, were applied as appropriate. Animal sample size was determined based on previous OSCC xenograft studies and our preliminary experiments, with consideration of detecting biologically meaningful differences in tumor growth while minimizing animal use. A two‐tailed *p*‐value of < 0.05 was considered statistically significant.

## 3 Results

### 3.1 DKK1 Expression Is Elevated in TCGA‐HNSC and Validated in OSCC Tissues

While elevated DKK1 expression has been documented in limited studies involving OSCC, its precise contribution to disease pathogenesis has yet to be elucidated. The TCGA‐HNSC analysis showed that DKK1 transcript levels were significantly increased in HNSC compared with adjacent non‐cancerous tissues (Figure 1A). Moreover, DKK1 expression was positively correlated with advanced clinical stage, histological grade, and lymph node metastasis (N stage) (Figure 1B). High DKK1 expression also correlated with worse OS in the cohort (HR = 2.190; 95% CI: 1.669–2.875; *p* < 0.0001) (Figure 1C). These findings were validated in a local cohort of 12 matched OSCC samples, where 10 cases exhibited elevated DKK1 mRNA and protein levels compared to paired normal tissues (Figure 1D,E). Immunohistochemical staining additionally corroborated the marked elevation of DKK1 in neoplastic tissues relative to matched healthy margins (Figure 1F). Collectively, these findings suggest that elevated DKK1 expression is associated with tumor progression and unfavorable survival in the TCGA‐HNSC cohort, and its upregulation was further supported by our OSCC tissue validation cohort.

**FIGURE 1:**
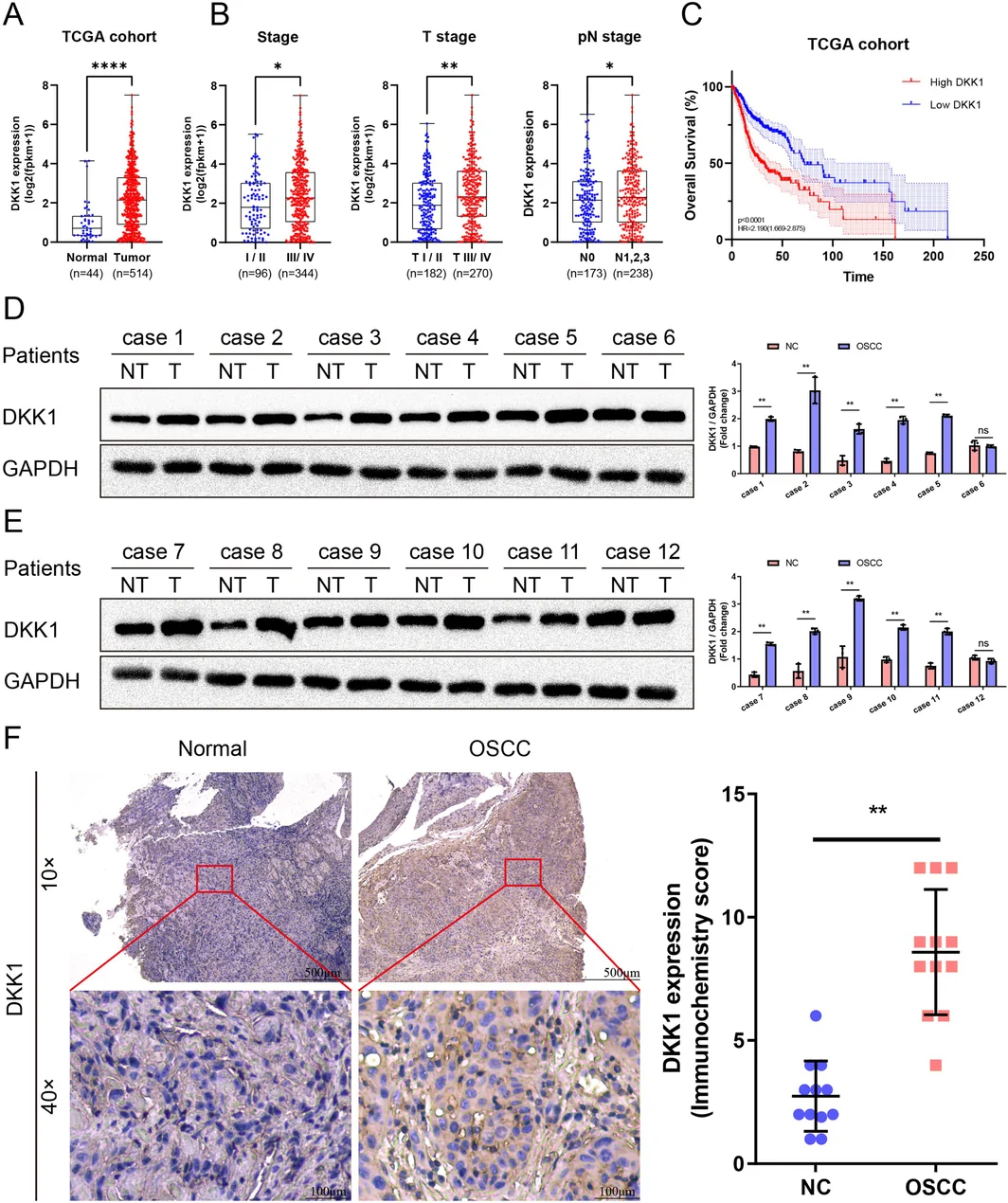
DKK1 expression is elevated in TCGA‐HNSC and validated in OSCC tissues. (A) Comparative analysis of DKK1 mRNA expression between tumor and adjacent normal tissues in the TCGA‐HNSC cohort. (B) Association of DKK1 expression with clinical stage, histological grade, and nodal status (N stage) in the TCGA‐HNSC cohort. (C) Kaplan–Meier analysis of overall survival (OS) in patients from the TCGA‐HNSC cohort stratified by DKK1 expression. (D, E) DKK1 mRNA (RT‐qPCR) and protein (western blot) expression in 12 paired OSCC and matched adjacent non‐tumor tissues. (F) Representative IHC staining of DKK1 in OSCC and matched adjacent non‐tumor tissues. Scale bars are indicated in the images. The corresponding scatter plot shows the IHC score distribution. For TCGA analyses, box plots show the median and interquartile range. Statistical significance was assessed using the Mann–Whitney *U* test for two‐group comparisons or Kruskal‐Wallis test for multiple‐group comparisons. Survival differences were analyzed using the log‐rank test. For paired OSCC tissue analyses, data are presented as the mean ± SD; *n* = 12 paired samples. Statistical significance was assessed using paired Student's *t*‐test for RT‐qPCR and western blot quantification, and Wilcoxon matched‐pairs signed‐rank test for IHC scores. *p < 0.05, **p < 0.01; ns, not significant.

### 3.2 Elevated DKK1 Drives Cisplatin Resistance in OSCC Cells

To elucidate the involvement of DKK1 in chemoresistance, we initially evaluated its expression profile in response to cisplatin challenge. Both transcriptional and translational analyses revealed that cisplatin stimulation triggered DKK1 upregulation in a dose‐proportional manner (Figure 2A,B). Subsequently, we established two refractory OSCC sublines (CAL‐27‐CR and SCC‐9‐CR) through prolonged drug exposure. Viability assays confirmed their resistant phenotypes, demonstrating substantially elevated IC50 thresholds (35.46 μM for CAL‐27‐CR and 47.11 μM for SCC‐9‐CR) relative to their respective wild‐type parental cells (CAL‐27, 17.15 μM; SCC‐9, 17.08 μM) (Figure 2C,D). To further validate the resistant phenotype beyond IC50 shifts, Annexin V/PI flow cytometry was performed after cisplatin treatment. Under exposure to 10 μM cisplatin, CAL‐27‐CR and SCC‐9‐CR cells exhibited markedly lower Annexin V/PI‐positive apoptotic cell fractions than their corresponding parental cells (Figure S1). These findings indicate that the resistant sublines are less sensitive to cisplatin‐induced apoptosis/cell death, further supporting the successful establishment of cisplatin‐resistant OSCC cell models.

**FIGURE 2:**
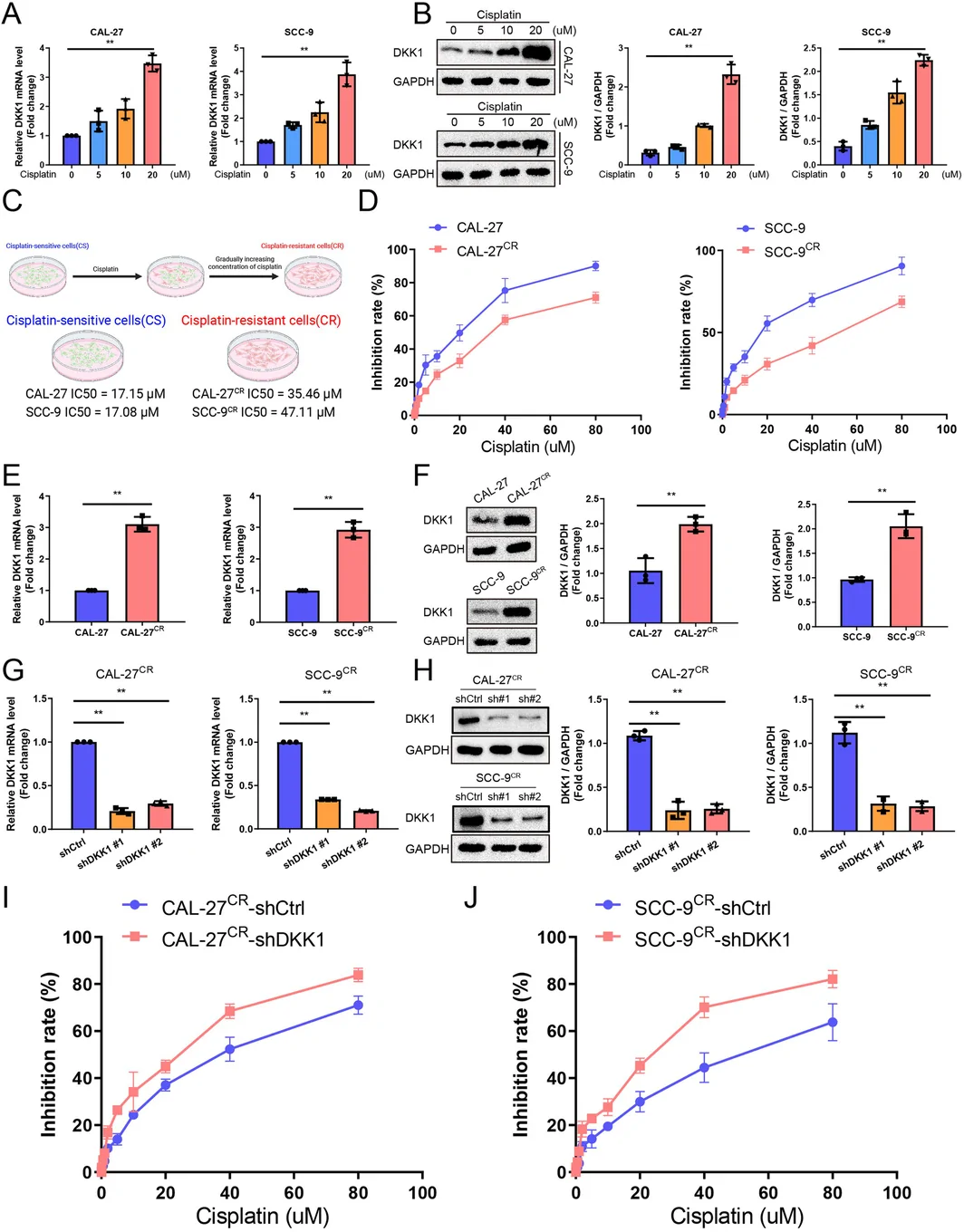
Elevated DKK1 contributes to cisplatin resistance in OSCC cells. (A, B) DKK1 mRNA and protein expression in CAL‐27 and SCC‐9 cells exposed to escalating doses of cisplatin (0–20 μM). (C) Schematic workflow illustrating the generation of cisplatin‐resistant (CR) cell lines. (D) CCK‐8 viability assays showing enhanced survival of CAL‐27‐CR and SCC‐9‐CR cells compared with their parental counterparts under 48‐h cisplatin treatment. (E, F) Baseline DKK1 mRNA and protein expression in CR cells relative to parental cells, detected by RT‐qPCR and western blotting. (G, H) DKK1 knockdown efficiency in CR cells transduced with shDKK1#1 or shDKK1#2 compared with shCtrl, assessed at both mRNA and protein levels. (I, J) Cell viability assays showing restored cisplatin sensitivity in CR cells following DKK1 silencing. Data are presented as the mean ± SD from three independent biological experiments unless otherwise indicated. Statistical significance was assessed using two‐tailed Student's *t*‐test for two‐group comparisons or one‐way ANOVA followed by Tukey's post hoc test for multiple‐group comparisons. For dose–response viability assays, IC50 values were calculated from nonlinear regression curves. *p < 0.05, **p < 0.01; ns, not significant.

Western blot analysis confirmed consistent DKK1 protein overexpression in both resistant cell lines (Figure 2E,F). To evaluate the functional role of DKK1, stable knockdown was performed in CAL‐27‐CR and SCC‐9‐CR cells using shRNA (Figure 2G,H). DKK1 silencing significantly restored cisplatin sensitivity compared to the shCtrl group (Figure 2I,J). These results indicate that DKK1 overexpression contributes to cisplatin resistance in OSCC, and that its suppression may provide a potential strategy to overcome therapeutic refractoriness.

### 3.3 DKK1 Knockdown Resensitizes OSCC Cells to Cisplatin via Ferroptosis Induction

To elucidate the mechanism underlying cisplatin‐induced cytotoxicity, cell death patterns were characterized via flow cytometry. Cisplatin exposure resulted in only a modest increase in the total cell death rate (including late apoptotic/necrotic populations) in both cisplatin‐resistant cells, with no significant alteration observed in early apoptotic fractions (Figure 3A). These observations indicate a prevalence of non‐apoptotic lethal pathways. To delineate the precise mechanism of cell death, the cultures were co‐treated with specific pharmacological agents targeting necroptosis (Nec‐1), apoptosis (Z‐VAD‐FMK), autophagy (CQ), and ferroptosis (Fer‐1). Notably, ferroptosis blockade via Fer‐1 was the sole intervention that effectively rescued cell viability in both chemoresistant lines, whereas the other antagonists proved ineffective at blunting cisplatin‐mediated toxicity (Figure 3B), implicating ferroptosis as the primary mechanism. Furthermore, the sensitivity of CAL‐27‐CR and SCC‐9‐CR cells to the ferroptosis inducer erastin was evaluated. Consistent with cisplatin resistance, both resistant lines were cross‐resistant to erastin (Figure 3C), supporting a connection between ferroptosis resistance and chemoresistance.

**FIGURE 3:**
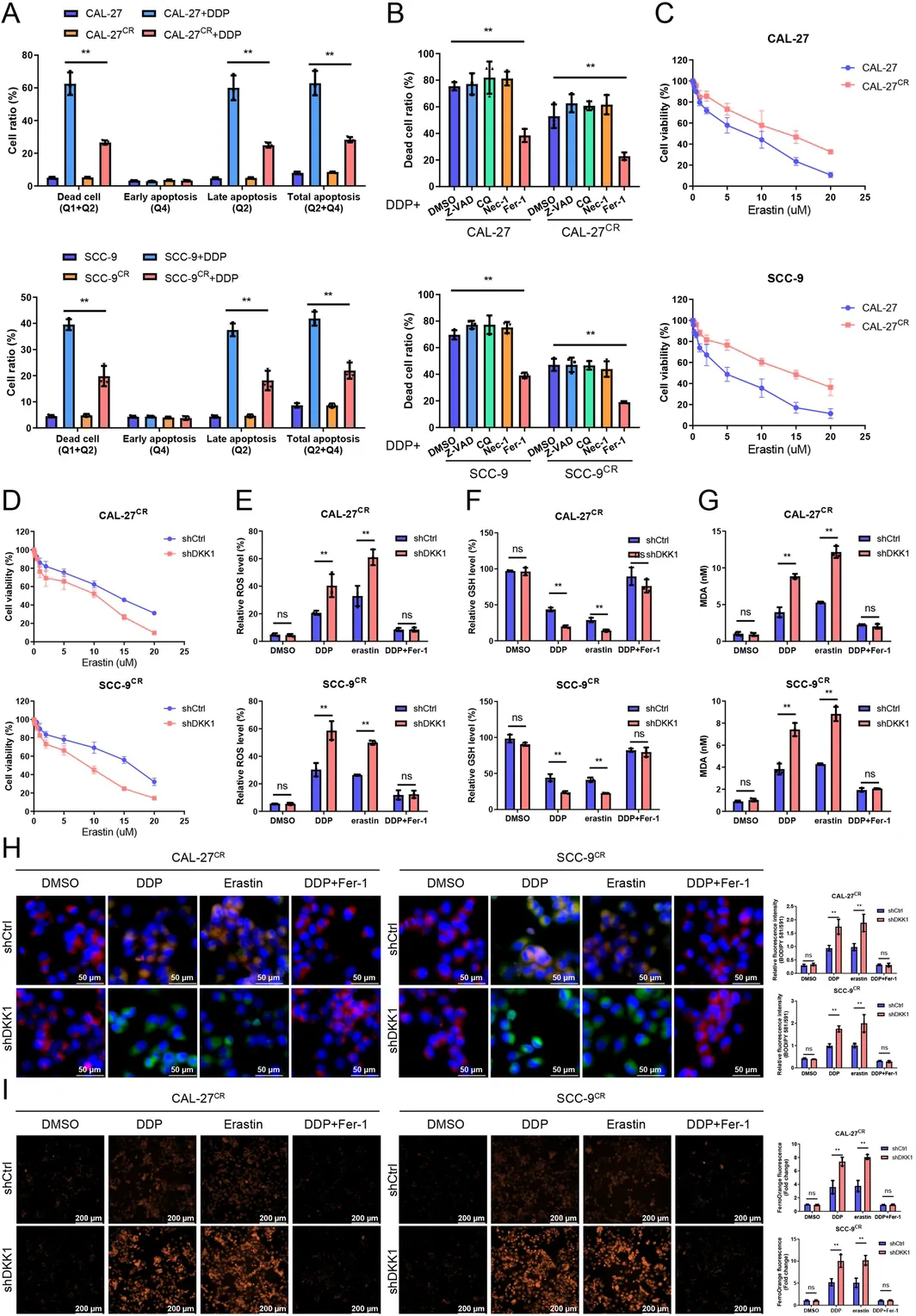
DKK1 knockdown resensitizes OSCC cells to cisplatin via ferroptosis induction. (A) Flow cytometric analysis using Annexin V‐FITC/PI staining to assess cell death in parental and CR cells after cisplatin (DDP) treatment. (B) Cell viability assessed by CCK‐8 assay in CR cells treated with DDP alone or combined with specific cell death inhibitors, including Nec‐1, Z‐VAD‐FMK, CQ, and Fer‐1, for 24 h. (C, D) Dose‐dependent effects of the ferroptosis inducer erastin on the viability of parental and CR cells (C), and DKK1‐depleted CR cells (D). (E–G) Quantification of ferroptosis‐associated markers, including relative ROS accumulation (E), GSH depletion (F), and MDA production (G), in shCtrl and shDKK1 CR cells subjected to DDP, erastin, or Fer‐1 treatment. (H) C11‐BODIPY 581/591 staining showing lipid peroxidation in shCtrl and shDKK1 CR cells subjected to DDP, erastin, or Fer‐1 treatment. (I) FerroOrange staining showing intracellular Fe^2+^ accumulation in shCtrl and shDKK1 CR cells subjected to DDP, erastin, or Fer‐1 treatment. Data are presented as the mean ± SD from three independent biological experiments unless otherwise indicated. Statistical significance was assessed using two‐tailed Student's *t*‐test for two‐group comparisons or one‐way ANOVA followed by Tukey's post hoc test for multiple‐group comparisons. For dose–response viability assays, IC50 values were calculated from nonlinear regression curves. *p < 0.05, **p < 0.01; ns, not significant.

To explore how DKK1 regulates ferroptosis in resistant OSCC, DKK1 was knocked down and oxidative stress markers were measured. DKK1 silencing significantly enhanced cisplatin and erastin‐induced ferroptotic responses, as indicated by increased ROS and MDA levels, and decreased GSH content and cell viability, which were notably reversed by co‐treatment with Fer‐1 (Figure 3D–G). Additional C11‐BODIPY and FerroOrange staining further showed DKK1 silencing significantly enhanced cisplatin and erastin‐induced lipid peroxidation and intracellular Fe^2+^ accumulation, and these effects were reversed by Fer‐1 (Figure 3H,I). These results suggest that DKK1 depletion restores cisplatin sensitivity by promoting ferroptosis, highlighting its pivotal role in regulating cell death in resistant OSCC.

### 3.4 DKK1 Silencing Triggers Ferroptosis Through the Downregulation of SLC3A2

To reveal the mechanism, we analyzed ferroptosis genes in resistant cells after stable DKK1 knockdown. The ferroptosis‐associated gene SLC3A2 (subunit of system Xc−) was strongly downregulated following DKK1 knockdown (Figure 4A). Correspondingly, SLC3A2 protein expression was reduced upon DKK1 depletion, as shown by Western blot analysis (Figure 4B). TCGA‐HNSC dataset analysis revealed significantly higher SLC3A2 expression in HNSC tissues compared to adjacent normal tissues (Figure 4C). Moreover, SLC3A2 expression levels were positively correlated with histological grade (Figure 4D), and high SLC3A2 expression was associated with worse OS (HR = 1.475; 95% CI: 1.128–1.929; *p* = 0.0046) (Figure 4E). DKK1 and SLC3A2 expression were strongly positively correlated in HNSC by bioinformatics analysis (Figure 4F). These results suggest a positive association between DKK1 and SLC3A2 in the TCGA‐HNSC cohort and support further functional investigation in OSCC cells.

**FIGURE 4:**
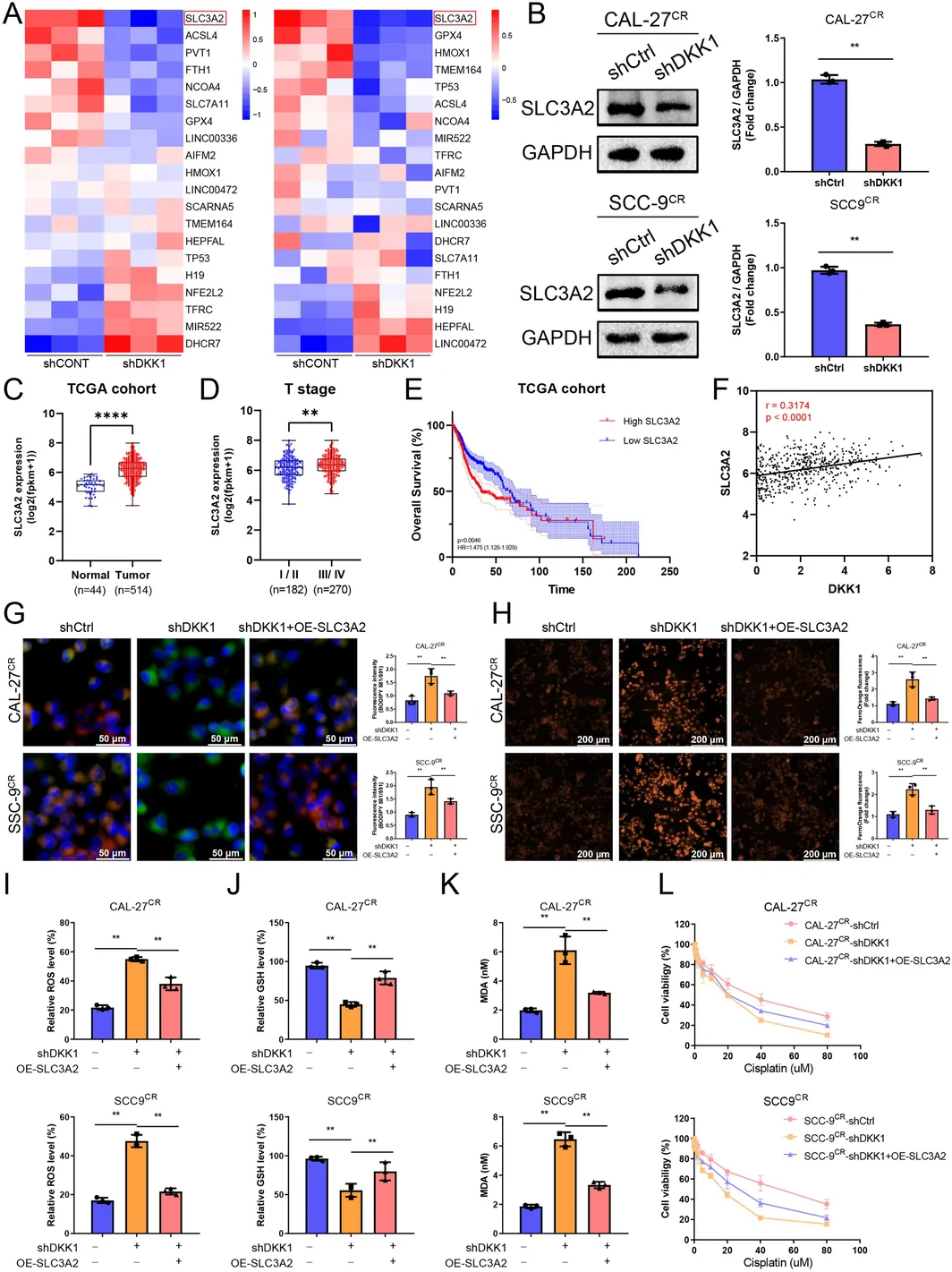
DKK1 silencing triggers ferroptosis through the downregulation of SLC3A2. (A, B) Expression profiles of SLC3A2 and DKK1 in CR cells following DKK1 depletion, evaluated by western blotting and RT‐qPCR. (C, D) TCGA‐HNSC analysis showing SLC3A2 mRNA expression in tumor and adjacent normal tissues (C) and its distribution across histological grades (D). (E) Kaplan–Meier analysis of overall survival (OS) in patients from the TCGA‐HNSC cohort stratified by SLC3A2 expression. (F) Pearson correlation analysis showing a positive correlation between DKK1 and SLC3A2 expression within the TCGA‐HNSC cohort. (G, H) Effects of SLC3A2 overexpression on C11‐BODIPY 581/591 oxidation (G) and FerroOrange fluorescence (H) in DKK1‐depleted CR cells. (I–K) Effects of SLC3A2 overexpression on ferroptosis‐associated markers, including ROS accumulation (I), GSH levels (J), and MDA production (K), in DKK1‐depleted CR cells. (L) CCK‐8 cell viability assay showing the effect of SLC3A2 overexpression on cisplatin sensitivity in DKK1‐depleted CR cells. Data are presented as the mean ± SD from three independent biological experiments unless otherwise indicated. For TCGA‐HNSC analyses, box plots show the median and interquartile range. Statistical significance was assessed using two‐tailed Student's *t*‐test for two‐group comparisons, one‐way ANOVA followed by Tukey's post hoc test for multiple‐group comparisons, Mann–Whitney *U* test or Kruskal‐Wallis test for TCGA‐HNSC group comparisons, and log‐rank test for survival analysis. Correlation was assessed using Pearson correlation analysis. *p < 0.05, **p < 0.01; ns, not significant.

To determine whether DKK1 downregulation promotes ferroptosis through SLC3A2 suppression, rescue experiments were performed by overexpressing SLC3A2 in DKK1‐knockdown cells (Figure S2A,B). Functional validation showed that C11‐BODIPY 581/591 staining demonstrated that DKK1 knockdown enhanced lipid peroxidation, whereas SLC3A2 overexpression markedly reduced oxidized C11‐BODIPY fluorescence (Figure 4G). FerroOrange staining further showed that SLC3A2 overexpression attenuated the intracellular Fe^2+^ accumulation induced by DKK1 depletion (Figure 4H). In addition, SLC3A2 overexpression effectively attenuated shDKK1‐induced ROS accumulation, reduced MDA production, and restored GSH levels (Figure 4I–K). Consistent with these ferroptosis‐related changes, SLC3A2 overexpression reversed the growth‐inhibitory effect of DKK1 knockdown and restored cisplatin resistance (Figure 4L). These findings support SLC3A2 as a key downstream mediator of DKK1‐associated ferroptosis suppression and cisplatin resistance.

### 3.5 DKK1 Modulates SLC3A2 via the WNT/β‐Catenin/STAT3 Signaling Cascade

The potential mechanism through which DKK1 upregulates SLC3A2 was further investigated. STAT3 has been reported to bind the promoter region of system Xc − components and repress their expression [31], and that the Wnt/β‐catenin pathway can enhance STAT3 activity [32]. To determine whether DKK1 regulates SLC3A2 expression through the β‐catenin/STAT3 axis, functional assays were conducted. Genetic ablation of DKK1 increased β‐catenin expression and STAT3 phosphorylation while reducing SLC3A2 protein levels (Figure 5A–D). Notably, these effects were attenuated by STAT3 inhibition, suggesting that STAT3 activity is involved in the regulation of SLC3A2 following DKK1 depletion.

**FIGURE 5:**
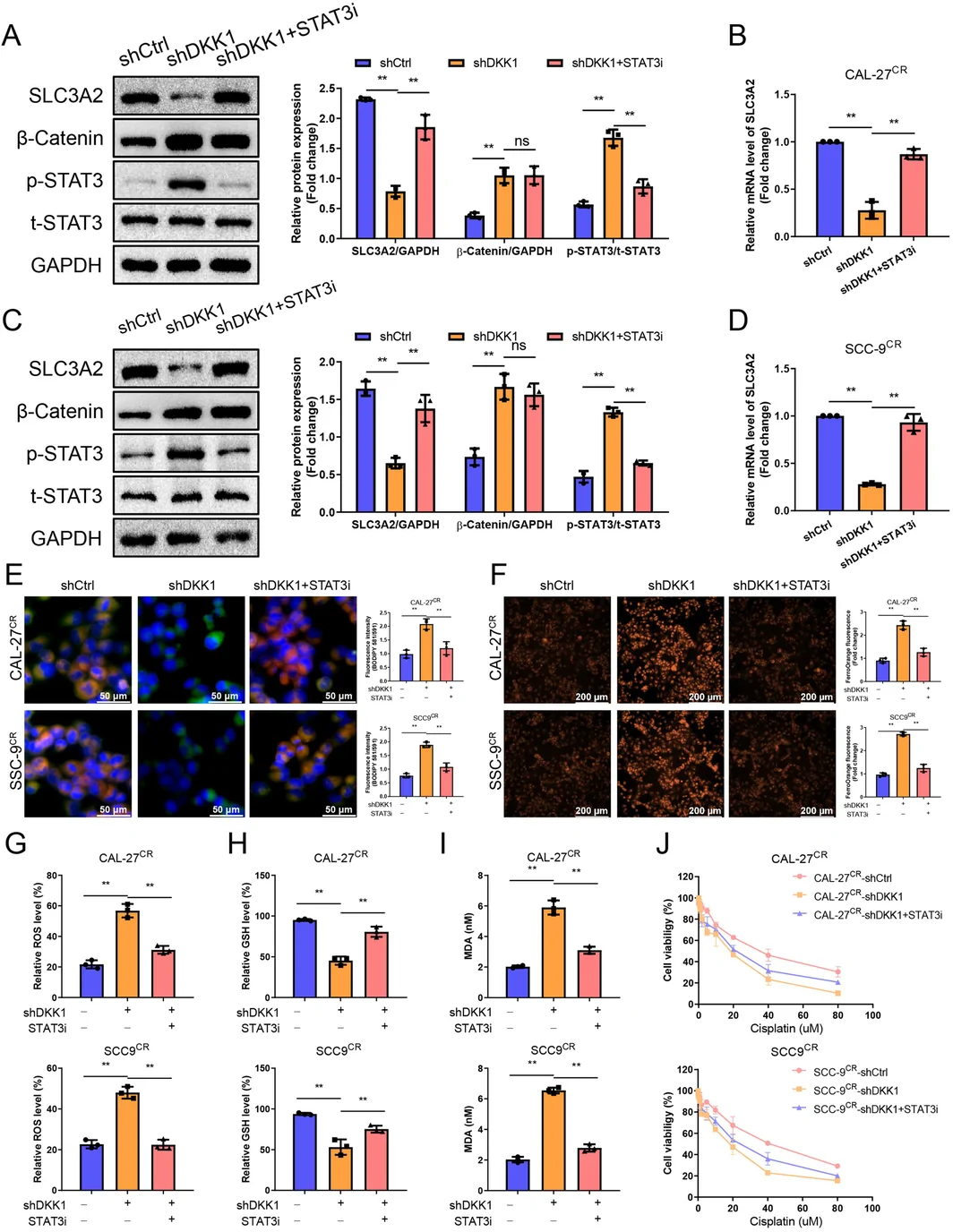
DKK1 regulates SLC3A2‐associated ferroptosis through the β‐catenin/STAT3 axis. (A‐D) Western blotting and RT‐qPCR analyses of pathway‐associated markers in CAL‐27‐CR (A, B) and SCC‐9‐CR (C, D) cells following DKK1 knockdown and/or treatment with the STAT3 inhibitor STAT3‐IN‐3 (1 μM, 24 h). (E) C11‐BODIPY 581/591 staining showing lipid peroxidation in CR cells following DKK1 knockdown and/or STAT3 inhibition. (F) FerroOrange staining showing intracellular Fe^2+^ accumulation in CR cells following DKK1 knockdown and/or STAT3 inhibition. (G–I) Assessment of ferroptosis‐associated indicators, including ROS accumulation (G), GSH levels (H), and MDA production (I), in CR cells following DKK1 knockdown and/or STAT3 inhibition. (J) CCK‐8 cell viability assay showing the effects of DKK1 knockdown and/or STAT3 inhibition on cisplatin sensitivity in CR cells. Data are presented as the mean ± SD from three independent biological experiments unless otherwise indicated. Statistical significance was assessed using two‐tailed Student's *t*‐test for two‐group comparisons or one‐way ANOVA followed by Tukey's post hoc test for multiple‐group comparisons. *p < 0.05, **p < 0.01; ns, not significant.

To further investigate whether STAT3 directly regulates SLC3A2 transcription, ChIP assays were performed. STAT3 binding was detected at the promoter region of SLC3A2, and ChIP‐qPCR analysis showed that DKK1 knockdown significantly enhanced STAT3 enrichment at the SLC3A2 promoter (Figure S3A,B). Consistently, SLC3A2 promoter luciferase reporter assays revealed that DKK1 knockdown significantly decreased SLC3A2 promoter activity, whereas STAT3 inhibition partially restored promoter activity in DKK1‐depleted cells (Figure S3C). These findings indicate that STAT3 directly participates in the transcriptional regulation of SLC3A2. We next performed β‐catenin loss‐ and gain‐of‐function experiments to clarify its upstream role in this pathway. In DKK1‐knockdown cells, β‐catenin silencing attenuated STAT3 phosphorylation and restored SLC3A2 expression (Figure S3D). Conversely, β‐catenin overexpression increased STAT3 phosphorylation and reduced SLC3A2 expression (Figure S3E). These results support β‐catenin as an upstream regulator of STAT3 activation and SLC3A2 suppression in this context.

Further investigation of ferroptosis‐related changes showed that STAT3 inhibition reduced oxidized C11‐BODIPY fluorescence and FerroOrange staining in DKK1‐depleted cells, indicating decreased lipid peroxidation and intracellular Fe^2+^ accumulation (Figure 5E,F). In addition, STAT3 inhibition attenuated shDKK1‐induced ROS and MDA accumulation and restored GSH levels toward baseline (Figure 5G–I). Moreover, STAT3 inhibition significantly reversed the chemosensitizing effect of DKK1 knockdown, partially reinstating cisplatin resistance in OSCC cells (Figure 5J). Collectively, these data support a signaling hierarchy in which DKK1 maintains SLC3A2 expression by restraining β‐catenin/STAT3‐mediated transcriptional repression, thereby contributing to ferroptosis resistance and cisplatin tolerance in OSCC cells.

### 3.6 DKK1 Depletion Potentiates Cisplatin Efficacy Against Resistant OSCC Xenografts in Vivo

To test the in vivo therapeutic potential of DKK1/SLC3A2 targeting for ferroptosis, CAL‐27‐CR xenografts in BALB/c nude mice were treated with cisplatin (2.5 mg/kg, twice weekly) for 4 weeks. DKK1 silencing enhanced the antitumor effect of cisplatin, as indicated by reduced tumor size, tumor weight, and tumor growth rate, whereas concomitant SLC3A2 overexpression largely reversed this inhibitory effect (Figure 6A–C). IHC analysis of xenograft tissues revealed that shDKK1 tumors exhibited reduced DKK1 expression, increased β‐catenin and p‐STAT3 levels, and downregulated SLC3A2 expression (Figure 6D). To further assess ferroptosis activation in vivo, 4‐HNE staining was performed as an indicator of lipid peroxidation. Tumors from the shDKK1 plus cisplatin group displayed markedly increased 4‐HNE staining compared with the shCtrl plus cisplatin group, while SLC3A2 overexpression attenuated this increase (Figure 6E). These findings provide additional in vivo evidence that DKK1 depletion enhances cisplatin efficacy, at least in part, by promoting SLC3A2‐associated lipid peroxidation and ferroptosis.

**FIGURE 6:**
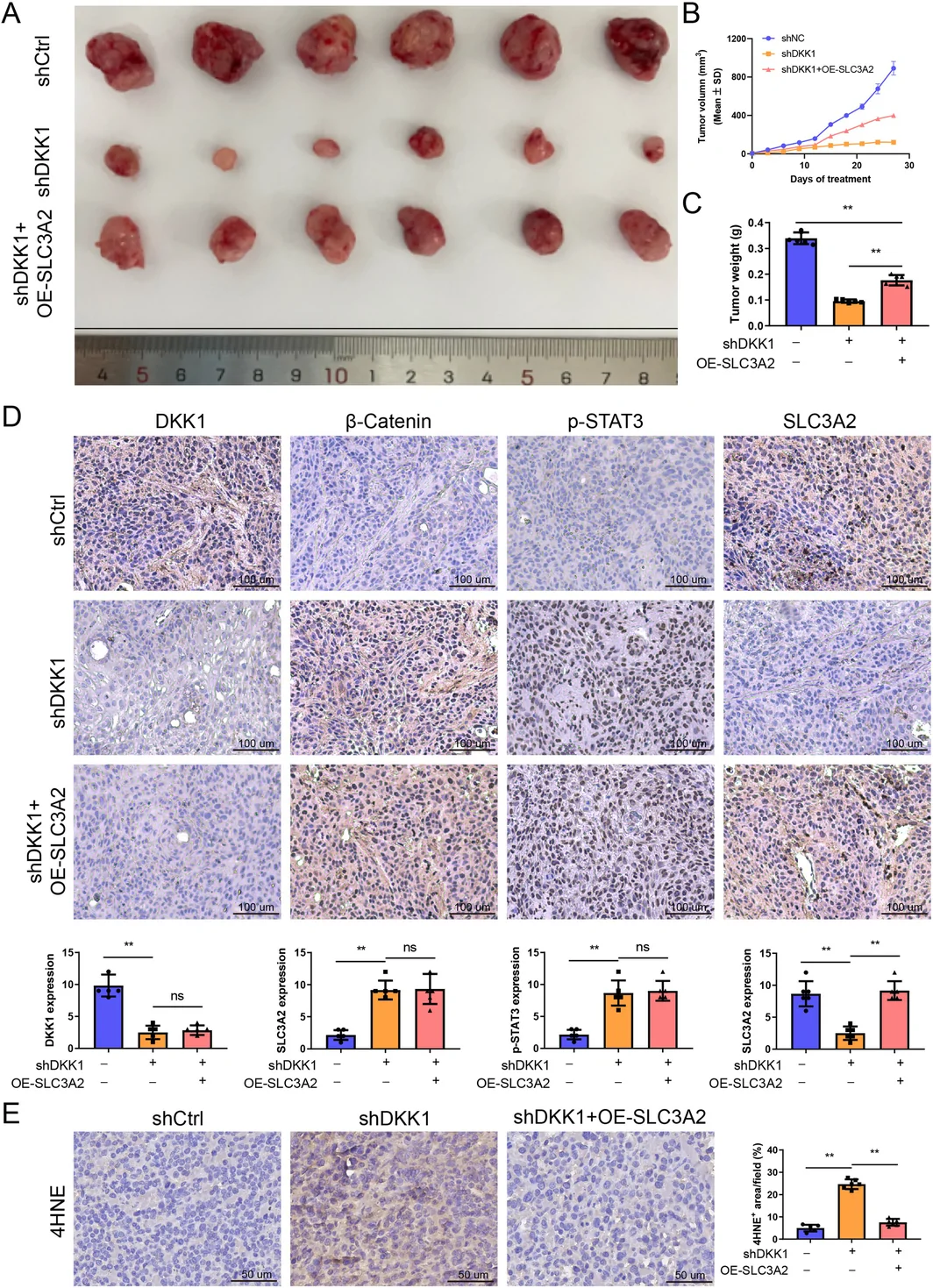
DKK1 depletion potentiates cisplatin efficacy against resistant OSCC xenografts in vivo. (A, B) Macroscopic appearance of excised tumors at day 27 post‐treatment (A) and the corresponding terminal tumor weights (B) across different cohorts. (C) Tumor volume growth trajectories in mice bearing CAL‐27‐CR xenografts subjected to different interventions. (D) Representative immunohistochemical (IHC) staining showing DKK1, β‐catenin, p‐STAT3, and SLC3A2 expression patterns in resected xenograft tissues. Scale bar = 100 μm. (E) Representative IHC staining of 4‐HNE in xenograft tissues, indicating lipid peroxidation in vivo. Scale bar = 50 μm. Data are presented as the mean ± SD; *n* = 6 mice per group. Statistical significance was assessed using one‐way ANOVA followed by Tukey's post hoc test for comparisons among multiple groups. Tumor growth curves were analyzed using repeated‐measures ANOVA. *p < 0.05, **p < 0.01; ns, not significant.

## 4 Discussion

This study systematically elucidated the molecular mechanism by which DKK1 confers cisplatin resistance in OSCC through the regulation of ferroptosis. Analysis of TCGA data and clinical patient samples revealed that DKK1 is significantly overexpressed in OSCC tissues, with high expression strongly correlating with poor OS, suggesting its potential as a prognostic biomarker. Further investigation demonstrated that DKK1 overexpression is linked to cisplatin resistance, a finding validated using cisplatin‐resistant OSCC cell lines. Mechanistically, DKK1 suppresses ferroptosis by upregulating SLC3A2, thereby promoting tumor cell survival. In contrast, DKK1 knockdown sensitized resistant cells to cisplatin and induced ferroptosis. Detailed pathway analysis revealed that DKK1‐mediated regulation of SLC3A2 is dependent on the Wnt/β‐catenin/STAT3 axis. Importantly, in vivo experiments confirmed that targeting DKK1 effectively reverses cisplatin resistance and suppresses tumor growth by enhancing ferroptosis. These findings not only establish DKK1 as a critical indicator of cisplatin resistance in OSCC but also highlight its therapeutic potential for overcoming chemotherapy resistance in clinical settings.

As a key Wnt pathway regulator, DKK1 plays critical roles in embryogenesis and cancer formation [33]. Previous studies have demonstrated that DKK1 is aberrantly expressed in various malignancies, including breast, colorectal, and esophageal cancers, and promotes tumor progression by modulating the TME, EMT, and stemness [26, 34, 35]. Emerging evidence suggests that DKK1 contributes to therapy resistance in cancer. For example, DKK1 has been shown to induce bortezomib resistance in multiple myeloma via the CKAP4 receptor and is associated with EGFR‐TKI resistance in non‐small cell lung cancer [36, 37]. Nevertheless, the involvement and mechanistic basis of DKK1 in OSCC chemoresistance to cisplatin remain unknown. To our knowledge, this study provides the first evidence that DKK1 expression is significantly elevated in cisplatin‐resistant OSCC cells compared to their parental controls. DKK1 inhibition or knockdown re‐sensitized resistant cells to cisplatin and suppressed their proliferation, thus establishing DKK1 as a key driver of cisplatin resistance. Beyond its established roles, our findings position DKK1 as a potential mediator of acquired chemoresistance in OSCC and highlight its potential as a therapeutic target for overcoming drug resistance.

Ferroptosis has emerged as an important mechanism linking redox homeostasis, metabolic vulnerability, and therapeutic response in OSCC. Recent OSCC‐focused studies and reviews suggest that ferroptosis is mainly controlled by coordinated regulation of iron metabolism, lipid peroxidation, and antioxidant defense systems, particularly the system Xc−/GSH/GPX4 axis [9, 10]. In the context of cisplatin resistance, resistant OSCC and head and neck cancer cells often acquire enhanced redox‐buffering capacity and reduced ferroptotic vulnerability, allowing them to survive chemotherapy‐induced oxidative stress [10, 11]. Consistently, pharmacological or genetic induction of ferroptosis has been shown to restore cisplatin sensitivity by targeting pathways such as SLC7A11/xCT‐mediated cystine transport, Nrf2/HO‐1/xCT signaling, AEBP1‐associated MAPK signaling, and circRNA‐miRNA‐SLC7A11 regulatory networks [11, 12, 13, 14]. These findings collectively support ferroptosis evasion as a recurrent adaptive mechanism in OSCC chemoresistance. In line with this concept, our study shows that DKK1 knockdown re‐sensitizes cisplatin‐resistant OSCC cells to ferroptosis, as evidenced by increased ROS and MDA levels, reduced GSH content, enhanced C11‐BODIPY oxidation, increased FerroOrange staining, and rescue by ferroptosis inhibition. Compared with previous studies that mainly focused on intracellular ferroptosis regulators such as SLC7A11/xCT, GPX4, Nrf2, or non‐coding RNA‐mediated regulation, our findings suggest that DKK1 may act as an upstream tumor‐promoting factor that maintains SLC3A2‐associated ferroptosis resistance. Because SLC3A2 is an essential component of system Xc− and supports cystine uptake and antioxidant defense, the DKK1/SLC3A2 axis may represent an additional regulatory layer linking extracellular oncogenic signaling to ferroptotic vulnerability and cisplatin response. This expands the current understanding of ferroptosis‐mediated chemoresistance in OSCC and provides a potential rationale for combining DKK1/SLC3A2‐targeted strategies with cisplatin‐based therapy.

Within the cellular antioxidant machinery, SLC3A2 operates as a core structural element of the system Xc− transporter, vital for sustaining redox homeostasis [38]. Recent reports indicate that SLC3A2 drives ferroptosis resistance in various cancers by regulating cystine uptake and glutathione synthesis [39, 40]. In breast and ovarian cancer, SLC3A2 overexpression has been closely linked to therapy resistance [41, 42], suggesting its potential as a therapeutic target. However, the regulatory mechanisms of SLC3A2 and its role in cisplatin resistance in OSCC remain incompletely understood. In this study, DKK1 knockdown reduced SLC3A2 expression and promoted ferroptosis‐associated changes, including increased ROS and MDA levels, reduced GSH content, enhanced C11‐BODIPY oxidation, and increased FerroOrange staining. Conversely, SLC3A2 overexpression attenuated these ferroptotic phenotypes and restored cisplatin resistance, supporting SLC3A2 as a key downstream mediator of DKK1‐associated ferroptosis suppression. Mechanistically, our additional ChIP, ChIP‐qPCR, and promoter luciferase assays showed that STAT3 binds to the SLC3A2 promoter, that DKK1 depletion enhances STAT3 enrichment at this promoter, and that STAT3 inhibition partially restores SLC3A2 promoter activity. β‐catenin loss‐ and gain‐of‐function experiments further placed β‐catenin upstream of STAT3 in this regulatory process. These findings support a model in which high DKK1 expression maintains SLC3A2 expression by restraining β‐catenin/STAT3‐mediated transcriptional repression. Conversely, DKK1 depletion releases β‐catenin/STAT3 signaling, suppresses SLC3A2 transcription, and promotes ferroptosis‐associated cisplatin sensitivity. This interpretation reconciles the classical role of DKK1 as a canonical Wnt/β‐catenin antagonist with its pro‐resistance function in cisplatin‐resistant OSCC cells.

Cisplatin remains a frontline therapy for advanced OSCC; however, the frequent emergence of resistance significantly limits its long‐term efficacy [6]. In vivo studies demonstrated that genetic inhibition of DKK1 markedly enhanced cisplatin sensitivity in resistant OSCC models. Combination therapy significantly suppressed tumor growth, reduced SLC3A2 expression, and reactivated ferroptosis, establishing ferroptosis restoration as the key mechanism to overcome resistance. Mechanistic rescue experiments further confirmed this pathway: Enforced SLC3A2 overexpression abolished the tumor‐suppressive effects of DKK1 inhibition, suggesting that DKK1 mediates cisplatin resistance by activating SLC3A2 to suppress ferroptosis. While no DKK1 inhibitor is yet clinically available, the monoclonal antibody DKN‐01 is under Phase II investigation, mainly for gastric and gynecologic cancers [43]. Therefore, targeting the DKK1/Wnt/β‐catenin/STAT3/SLC3A2 axis represents a promising and potential strategy for OSCC. DKK1 upregulation or ferroptosis impairment might serve as predictors for identifying responders to this therapy. A schematic overview illustrating how DKK1 blockade restores cisplatin efficacy by promoting ferroptosis through SLC3A2 downregulation is provided in Figure 7.

**FIGURE 7:**
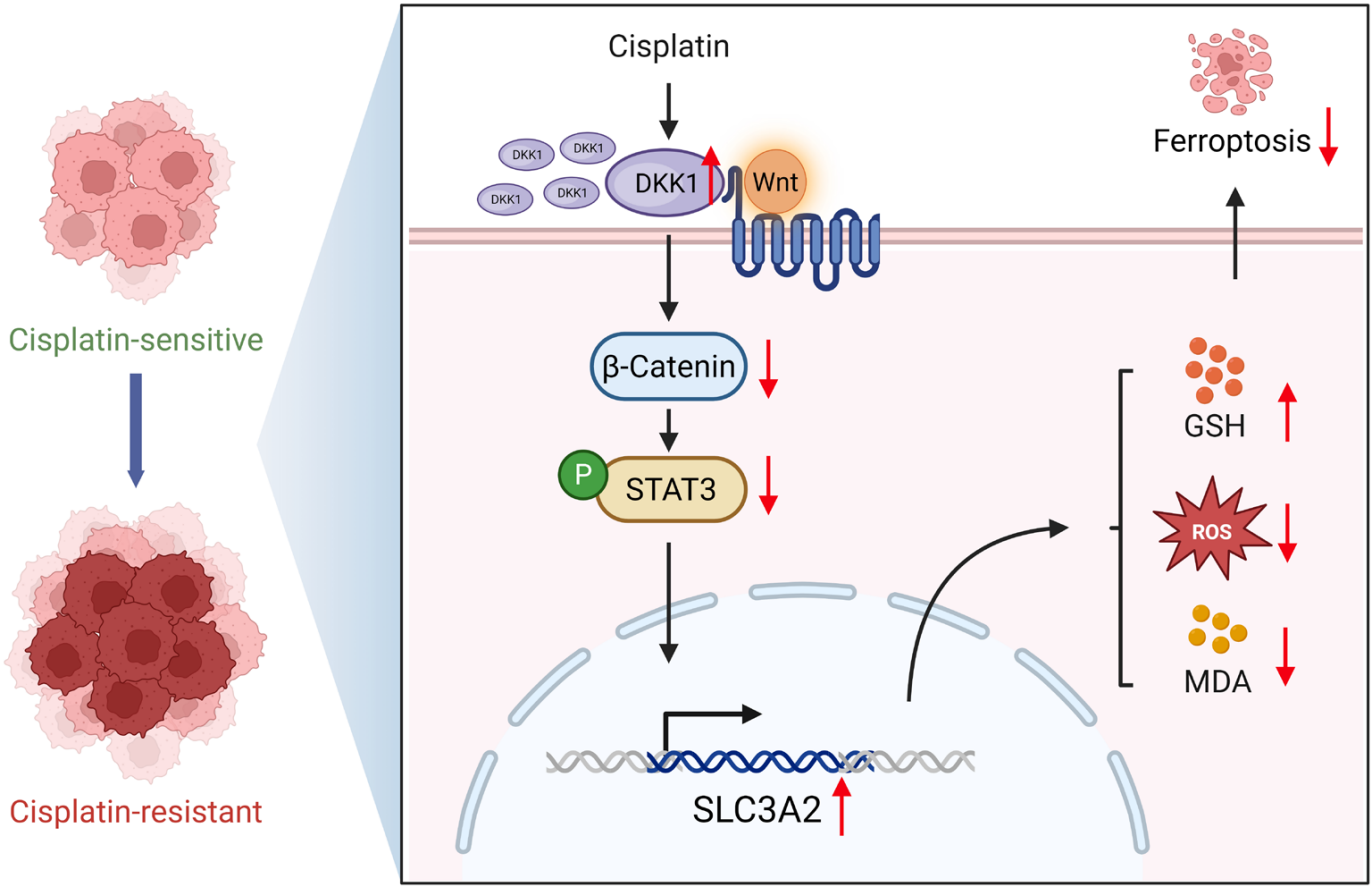
Graphical abstract. DKK1 Confers Cisplatin Resistance in Oral Squamous Cell Carcinoma via WNT/β‐catenin/STAT3‐SLC3A2‐Mediated Ferroptosis Suppression.

Despite providing mechanistic insights into the role of DKK1 in cisplatin resistance, this study has several limitations. First, the regulation of ferroptosis is highly complex, and the study primarily focused on the DKK1/SLC3A2 axis mediated through Wnt/β‐catenin/STAT3 signaling, without comprehensively exploring additional regulatory pathways or alternative ferroptotic modulators. Second, while TCGA analysis and patient samples confirmed DKK1 overexpression and its prognostic relevance, the clinical cohorts were relatively small, and larger, independent patient populations are needed to validate its utility as a biomarker. Third, proof of concept from resistant lines and xenografts requires validation with PDX and GEMM models to better replicate TME and therapy responses. Finally, the role of the TME in DKK1‐mediated resistance should be considered. Recent studies suggest that cancer‐associated fibroblasts (CAFs) can secrete DKK1 in response to chemotherapeutic stress, promoting an immunosuppressive and drug‐resistant niche [37, 44]. Thus, paracrine DKK1 from the stroma may act synergistically with autocrine DKK1 to sustain SLC3A2 expression and ferroptosis resistance in OSCC, a mechanism that warrants further investigation.

In conclusion, this study demonstrates that DKK1 drives cisplatin resistance in OSCC by upregulating SLC3A2 via the Wnt/β‐catenin/STAT3 axis, thereby inhibiting ferroptosis. DKK1 inhibition promotes ferroptosis by downregulating SLC3A2, enhancing cisplatin efficacy. Thus, targeting the DKK1/Wnt/β‐catenin/STAT3/SLC3A2 axis emerges as a novel therapeutic strategy for drug‐resistant oral cancer, potentially in combination with existing chemotherapies.

## Author Contributions

**Zhuoxuan Liu:** conceptualization, methodology, formal analysis, writing – original draft, project administration, validation, visualization. **Xiaolan Chen:** conceptualization, resources, data curation, software, validation. **Yucong Lin:** funding acquisition, investigation. **Lijun Zhu:** methodology, supervision, writing – original draft, writing – review and editing.

## Funding

This work was supported by the Postdoctoral Foundation from the Ministry of Human Resources and Social Security of China (Grant No. 110215901). The authors sincerely appreciate the financial support for this research.

## Ethics Statement

All the animal experiments were performed following the policies of the Animal Ethics Committee of Guangdong Provincial People's Hospital and conducted in strict compliance with ethical guidelines. Following the Declaration of Helsinki, this study acquired the approval by the Medical Ethics Committee of Guangdong Provincial People's Hospital. Informed consent was acquired from all the patients. Approval number: KY2025‐737‐02.

## Conflicts of Interest

The authors declare no conflicts of interest.

## Supporting information


**Figure S1:** Additional validation of cisplatin‐resistant OSCC cell lines. (A, B) Annexin V/PI flow cytometry analysis of apoptosis/cell death in parental and cisplatin‐resistant CAL‐27 (A) and SCC‐9 (B) cells following treatment with 10 μM cisplatin. CAL‐27‐CR and SCC‐9‐CR cells showed lower Annexin V/PI‐positive cell fractions than their corresponding parental cells under the same cisplatin exposure. Data are presented as the mean ± SD from three independent biological experiments. Statistical significance was assessed using two‐tailed Student's *t*‐test. *p < 0.05, **p < 0.01.
**Figure S2:** Validation of SLC3A2 overexpression efficiency. (A, B) SLC3A2 overexpression efficiency in CAL‐27‐CR (A) and SCC‐9‐CR (B) cells was detected by western blotting after lentiviral transduction. Data are presented as the mean ± SD from three independent biological experiments. Statistical significance was assessed using two‐tailed Student's *t*‐test. **p < 0.01.
**Figure S3:** Mechanistic validation of the β‐catenin/STAT3/SLC3A2 regulatory axis. (A) ChIP assay showing STAT3 binding at the promoter region of SLC3A2 in CAL‐27‐CR cells. (B) ChIP‐qPCR analysis of STAT3 enrichment at the SLC3A2 promoter in shCtrl and shDKK1 CAL‐27‐CR cells. (C) SLC3A2 promoter luciferase reporter assay in CAL‐27‐CR cells following DKK1 knockdown and/or treatment with the STAT3 inhibitor STAT3‐IN‐3. (D) Western blot analysis of p‐STAT3 and SLC3A2 expression in DKK1‐knockdown CAL‐27‐CR cells transfected with si‐NC or si‐β‐catenin. (E) Western blot analysis of p‐STAT3 and SLC3A2 expression in CAL‐27‐CR cells transfected with vector control or β‐catenin overexpression plasmid. Data are presented as the mean ± SD from three independent biological experiments unless otherwise indicated. Statistical significance was assessed using two‐tailed Student's *t*‐test for two‐group comparisons or one‐way ANOVA followed by Tukey's post hoc test for multiple‐group comparisons. *p < 0.05, **p < 0.01; ns, not significant.
**Table SI:** Patient characteristics.
**Table SII:** Primers used for RT‐qPCR.
**Table SIII:** Antibodies used for Western Blot.

## Data Availability

The data that support the findings of this study are available from the corresponding author upon reasonable request.
