# Supplementary material for: DKK1 Promotes Cisplatin Resistance in Oral Squamous Cell Carcinoma via SLC3A2‐Dependent Ferroptosis Suppression
Source: Cancer Rep (Hoboken). 2026 Oct 8;9(10):e70715. doi: 10.1002/cnr2.70715 (PMC13649886; doi:10.1002/cnr2.70715)
Supplement: Supplementary file 1 [file CNR2-9-e70715-s001.docx]

**Supplementary materials for**

**DKK1 Promotes Cisplatin Resistance in Oral Squamous Cell Carcinoma *via* SLC3A2-Dependent Ferroptosis Suppression**

Zhuoxuan Liu^1,#^, Xiaolan Chen^2,#^, Yucong Lin^3,#^, Lijun Zhu^1,*^

Correspondence: zhulijun@gdph.org.cn(Lijun Zhu)

**This file includes:**

Supplementary Figure S1-3

Supplementary Tables SI-III

**Supplementary Figures**

**Supplementary FIGURE S1** Additional validation of cisplatin-resistant OSCC cell lines. (A, B) Annexin V/PI flow cytometry analysis of apoptosis/cell death in parental and cisplatin-resistant CAL-27 (A) and SCC-9 (B) cells following treatment with 10 μM cisplatin. CAL-27-CR and SCC-9-CR cells showed lower Annexin V/PI-positive cell fractions than their corresponding parental cells under the same cisplatin exposure. Data are presented as the mean ± SD from three independent biological experiments. Statistical significance was assessed using two-tailed Student’s t-test. *P < 0.05, **P < 0.01.

**Supplementary FIGURE S2** Validation of SLC3A2 overexpression efficiency. (A, B) SLC3A2 overexpression efficiency in CAL-27-CR (A) and SCC-9-CR (B) cells was detected by western blotting after lentiviral transduction. Data are presented as the mean ± SD from three independent biological experiments. Statistical significance was assessed using two-tailed Student’s t-test. **P < 0.01.

**Supplementary FIGURE S3** Mechanistic validation of the β-catenin/STAT3/SLC3A2 regulatory axis. (A) ChIP assay showing STAT3 binding at the promoter region of SLC3A2 in CAL-27-CR cells. (B) ChIP-qPCR analysis of STAT3 enrichment at the SLC3A2 promoter in shCtrl and shDKK1 CAL-27-CR cells. (C) SLC3A2 promoter luciferase reporter assay in CAL-27-CR cells following DKK1 knockdown and/or treatment with the STAT3 inhibitor STAT3-IN-3. (D) Western blot analysis of p-STAT3 and SLC3A2 expression in DKK1-knockdown CAL-27-CR cells transfected with si-NC or si-β-catenin. (E) Western blot analysis of p-STAT3 and SLC3A2 expression in CAL-27-CR cells transfected with vector control or β-catenin overexpression plasmid. Data are presented as the mean ± SD from three independent biological experiments unless otherwise indicated. Statistical significance was assessed using two-tailed Student’s t-test for two-group comparisons or one-way ANOVA followed by Tukey’s post hoc test for multiple-group comparisons. *P < 0.05, **P < 0.01; ns, not significant.

**Supplementary Tables**

**Supplementary Table SI. Patient characteristics**

| Characteristic | Number | % |
| --- | --- | --- |
| Age (median), years | 65(41-83) |  |
| Sex |  |  |
| Male | 7 | 58.3 |
| Female | 5 | 41.7 |
| Site |  |  |
| Tongue | 8 | 66.7 |
| Mandibular | 4 | 33.3 |
| T Stage |  |  |
| TI/TII | 6 | 50 |
| TIII-IV | 6 | 50 |
| N Stage |  |  |
| N0 | 9 | 75 |
| N1-3 | 3 | 25 |
| Metastasis |  |  |
| No | 12 | 100 |
| Yes | 0 | 0 |

**Supplementary Table SII. Primers used for RT-qPCR**

| **Gene** |  | **Primer sequence (5’-3’)** |
| --- | --- | --- |
| *DKK1(Human)* | Forward | CCTTGAACTCGGTTCTCAATTCC |
|  | Reverse | CAATGGTCTGGTACTTATTCCCG |
| *GPX4(Human)* | Forward | GAGGCAAGACCGAAGTAAACTAC |
|  | Reverse | CCGAACTGGTTACACGGGAA |
| *ACSL4(Human)* | Forward | CATCCCTGGAGCAGATACTCT |
|  | Reverse | TCACTTAGGATTTCCCTGGTCC |
| *SLC3A2(Human)* | Forward | TGAATGAGTTAGAGCCCGAGA |
|  | Reverse | GTCTTCCGCCACCTTGATCTT |
| *SLC7A11(Human)* | Forward | TCTCCAAAGGAGGTTACCTGC |
|  | Reverse | AGACTCCCCTCAGTAAAGTGAC |
| *NCOA4(Human)* | Forward | GAGGTGTAGTGATGCACGGAG |
|  | Reverse | GACGGCTTATGCAACTGTGAA |
| *AIFM2(Human)* | Forward | AGACAGGGTTCGCCAAAAAGA |
|  | Reverse | CAGGTCTATCCCCACTACTAGC |
| *HMOX1(Human)* | Forward | AAGACTGCGTTCCTGCTCAAC |
|  | Reverse | AAAGCCCTACAGCAACTGTCG |
| *TMEM164(Human)* | Forward | TCACTTCAGCGTCTTGCAGAT |
|  | Reverse | GCCGTAGAATGGGTCTGAGAT |
| *TP53(Human)* | Forward | AACTGCGGGACGAGACAGA |
|  | Reverse | AGCTTCAAGAGCGACAAGTTTT |
| *NFE2L2(Human)* | Forward | TCAGCGACGGAAAGAGTATGA |
|  | Reverse | CCACTGGTTTCTGACTGGATGT |
| *DHCR7(Human)* | Forward | GCTGCAAAATCGCAACCCAA |
|  | Reverse | GCTCGCCAGTGAAAACCAGT |
| *GAPDH(Human)* | Forward | TGTGGGCATCAATGGATTTGG |
|  | Reverse | ACACCATGTATTCCGGGTCAAT |

**Supplementary Table SIII. Antibodies used for Western Blot**

| **Antibody** | **Species** | **Source** | **Catalog** | **Dilution** | **Application** |
| --- | --- | --- | --- | --- | --- |
| *Anti-DKK1* | *R* | Proteintech | 21112-1-AP | WB: 1:1000  IHC: 1:200 | WB, IHC |
| *Anti-SLC3A2* | *R* | Abcam | ab307587 | WB: 1:1000  IHC: 1:5000 | WB, IHC |
| *Anti-beta-catenin* | *R* | Proteintech | 51067-2-AP | WB: 1:1000  IHC: 1:1000 | WB, IHC |
| *Anti-STAT3* | *R* | Proteintech | 60199-1-Ig | WB: 1:3000  IHC: 1:400 | WB, IHC, ChIP |
| *Anti-phospho-STAT3* | *R* | Cell Signaling Technology | D3A7 | WB: 1:1000  IHC: 1:200 | WB, IHC |
| *Anti-4-HNE* | *M* | Proteintech | 68538-1-Ig | IHC: 1:1000 | IHC |
| *Anti-GAPDH* | *R* | Cell Signaling Technology | 2118 | WB:  1:1000 | WB |
